# Efficacy of combination of venetoclax with azacitidine or chemotherapy in refractory/relapse acute leukemias of ambiguous lineage, not otherwise specified

**DOI:** 10.1186/s40164-021-00239-w

**Published:** 2021-09-16

**Authors:** Kaiqi Liu, Yan Li, Shaowei Qiu, Chunlin Zhou, Shuning Wei, Dong Lin, Guangji Zhang, Hui Wei, Ying Wang, Bingcheng Liu, Xiaoyuan Gong, Qiuyun Fang, Yang Song, Huijun Wang, Chengwen Li, Qinghua Li, Lihua Wu, Benfa Gong, Yuntao Liu, Jianxiang Wang, Yingchang Mi

**Affiliations:** State Key Laboratory of Experimental Hematology, National Clinical Research Center for Blood Diseases, Institute of Hematology & Blood Diseases Hospital, Chinese Academy of Medical Sciences & Peking Union Medical College, Tianjin, 300020 China

**Keywords:** Efficacy, venetoclax, ALAL-NOS, Relapse, Refractory

## Abstract

Acute leukemias of ambiguous lineage, not otherwise specified (ALAL-NOS) is a rare type of acute leukemia. Management of relapse/refractory (R/R) patients is still challenging.traditional chemotherapy treatment is not effective. In this paper, we reported 6 R/R patients diagnosed as ALAL-NOS in our hospital, who were treated with venetoclax based treatment (venetoclax combining with azacitidine or chemotherapy). All 6 patients achieved CR. Five of the six patients received allo-HSCT, four patients were still alive in CR until the follow-up day. Our data provide preliminary evidence and show that venetoclax based regimens are effective and safety in patients with R/R ALAL-NOS.

**Supplementary Information:**

The online version contains supplementary material available at 10.1186/s40164-021-00239-w.

## Letter to the editor

Acute leukemias of ambiguous lineage, not otherwise specified (ALAL-NOS) is an extremely rare type of acute leukemia. The induction therapy for ALAL-NOS often refers to ALAL treatment. The outcome of ALAL (especially mixed phenotype acute leukemia, MPAL) was improved by acute lymphoblastic leukemia (ALL)-like therapy and allogeneic hematopoietic stem cell transplantation (allo-HSCT) during the first complete remission (CR1) [1–6]. However, salvage treatment of relapse/refractory (R/R) ALAL-NOS remain major concerns. Allo-HSCT is the only available cure for ALAL-NOS, but the outcome remains poor if the patient did not achieve CR before allo-HSCT [6]. Traditional chemotherapy is not effective. Therefore, other salvage therapies are needed to improve the CR before allo-HSCT.

Venetoclax, a potent oral inhibitor of BCL-2, has demonstrated clinical efficacy in various hematological malignancies [7, 8]. It can be combined with hypomethylating agents (HMAs), low-dose cytarabine (LDAC) and dose-reduced IA (idarubicin + Ara-c) in the treatment of newly diagnosed acute myeloid leukemia (AML), and achieve ideal effect [9–11]. Some case reports showed that venetoclax was effective in mixed phenotype acute leukemia (MPAL) [12]. Herein, we evaluated the therapeutic effect of venetoclax-based treatment in six R/R ALAL-NOS patients at our institution.

Between September 2017 and July 2020, six R/R ALAL-NOS patients at our hospital, who fulfilled the criteria of ALAL-NOS according to the 2016 World Health Organization (WHO) classification, were treated with venetoclax-based regimen (venetoclax combined with azacitidine or chemotherapy). The diagnosis was based on morphology, cytochemistry, immunophenotype, cytogenetic and molecular results. Fluorescence in situ hybridization (FISH) analysis for BCR-ABL, p53 deletion and MLL rearrangements were performed at the time of diagnosis. Data of the six patients was collected and analyzed, including clinical characteristics, cytogenetics, gene mutation profile, prior therapy, complete remission (CR) rate, overall survival (OS, survival duration was defined from diagnosis of the disease to dead).

Characteristics of the six patients are shown in Table 1. There were two women and four men, with a median age of 26 years (range, 16–66). Normal karyotype was found in three cases. Abnormalities involving MLL gene at the 11q23 locus were found in one case by FISH. Mutational analysis by next-generation sequencing showed mutations such as PHF6, NOTCH1, FAT1, BCOR, IKZF1, PTPN11, NRAS, etc., which were commonly seen in ALL (especially in T-ALL); mutations associated with AML such as FLT3/ITD, TET2, DNMT3A, NPM1, IDH2 and IDH1 were not found. One patient (patient 1) had RUNX1 mutation. The immunophenotype by FACS and cytochemical staining of the 6 ALAL-NOS patients was listed in (Additional file 1: Table S1).

**Table 1:** Clinical characteristics at diagnosis of the 6 R/R ALAL-NOS patients

No	Sex	Age (y)	Diagnosis	WBC (10^9^/L)	BM blast cell(%)	Chromosome	Fish	Gene mutation(NGS)
1	F	66	ALAL-NOS	3.37	87.0	46,XX	Negative	RUNX1, FAT1, BCOR
2	M	16	ALAL-NOS	25.18	91.50	46, XY, t(10; 12)(p11.2; q15), inc[4]/ 46, XY	Negative	PHF6, NRAS, RAD21, CALR
3	M	23	ALAL-NOS	62.9	83.5	46,XY	Negative	PHF6 ; IKZF1 ; KMT2D ;WT1;PTPN11;EED
4	M	29	ALAL-NOS	2.36	68.0	46,XY	MLL(+)	IL7R
5	F	52	ALAL-NOS	5.57	73.5	46 XX,del(22)(q13)[3]/ 46,XX,del(8)(p21),del(22)(q13)[15]/ 46,XX,t(3;6),del(8)(p21),del(22)(q13)[1]/ 46,XX[1]	Negative	BCOR
6	M	22	ALAL-NOS	55.5	79.0	47,XY,del(5)(q31),del(13)(?q12q22),+mar[20]	Negative	NOTCH1 KMT2C PTPN11 TP53

Patient 1 relapsed after allo-HSCT and patients 2–6 were refractory to at least one cycle traditional induction chemotherapy. All six patients were re-induced with venetoclax-based regimen (Table 2). Venetoclax was initiated at 100 mg daily and ramped up to 400 mg daily within three days, with continuous administration for 28 days. AZA was subcutaneously administered at 75 mg/M^2^ daily for seven days in patient 1. She achieved CR after one course of treatment,without another allo-HSCT, patient relapsed after CR for nine months, and died of disease progression. The other five refractory patients were treated with venetoclax combined with chemotherapy (detailed chemotherapy regimens are shown in Table 2). Venetoclax was initiated at 100 mg daily and ramped up to 200–400 mg daily within three days, chemotherapy was started on the fourth day (venetoclax dose was 200 mg/day due to combination with voriconazole for patient 4). Venetoclax was administered for ≥ 14 days. After one course of treatment, three patients (patients 2, 5 and 6) achieved CR; one patient (patient 4) achieved CRi; one patient (patient 3) achieved PR. Patient 3 was retreated with the same regimen plus decitabine and subsequently achieved CR. Patients 2–6 received allo-HSCT after achieving CR. On May 1, 2021, the median follow-up time was 8.5 months (range, 5–36). Four patients remained in CR; while one patient (patient 3) died of 4-degree GVHD after allo-HSCT.

**Table 2:** Initial treatment, salvage therapy and outcome of 6 R/R ALAL-NOS patients

No.	Previous treatment	Reinduction treatment	VEN dose, (mg)	VEN days	Response to VEN combination	HSCT	Outcome	OS(m)
1	Chemotherapy and HSCT	VEN + AZA	400 mg	28d	CR	N	Dead	36
2	VDCP NR; DAC + HACVP NR	VEN + AAVP	400 mg	14d	CR	Y	CR	15
3	VDCP NR; DAC + HACVP NR	VEN + AAVP PR; DAC + VEN + AAVP	400 mg	21d	CR	Y	Dead	9
4	DAC + AAVP NR	VEN + HOAP	200 mg (with voriconazole)	14d	CRi	Y	CR	8
5	VDCP + AA NR	VEN + CA	400 mg	14d	CR	Y	CR	7
6	AACVP NR	VEN + HOAP	400 mg	14d	CR	Y	CR	5

VEN, venetoclax; DAC, decitabine; AZA, azacitidine; VCR, vincristine; DNR, daunorubincin; CTX, cyclophosphamide; Pred, prednisone; HHT, homoharringtonine; Ara-c, cytarabine; Acla, aclarubicin. VDCP:VCR + DNR + CTX + Pred; HACVP, HHT + Ara-c + CTX + VCR + Pred; AAVP, Ac,a + Ara-c + VCR + Pred; AA, Acla + Ara-c; CA cladribine + LDAC; AACVP, Acla + Ara-c + CTX + VCR + Pred; HOAP: HHT + Ara-c + VCR + Pred; HSCT, hematopoietic stem cell transplantation

No tumor lysis was observed. The most common non-hematological adverse events were Grade 2/3 nausea and vomiting. All the patients had Grade 3/4 hematological toxicity, including leucopenia, neutropenia and thrombocytopenia. However, no patient had venetoclax dose interruptions, or experienced major bleeding or serious infections.

Treatment of ALAL-NOS remains a challenge, especially for relapse/refractory patients. Traditional induction chemotherapy is ineffective. In this report, six R/R ALAL-NOS patients were treated with venetoclax-based therapy (combined with azacitidine or chemotherapy). All six patients achieved CR. Five of the six patients received allo-HSCT, of which four patients remained alive in CR at the final follow-up, one patient died of serious GVHD after transplantation. Patient 1 who did not receive allo-HSCT,died of disease progression.

Although the number of patients in this study was limited, the data provided preliminary evidence that venetoclax-based regimens are effective and safe in patients with refractory or relapsed ALAL-NOS. Venetoclax-based regimens should be tested and verified as high-efficacy initial induction therapy for ALAL-NOS.

## Supplementary Information

**Additional file 1: Table S1.** The immunophenotype by FACS and cytochemical staining of the ALAL-NOS patients. **Table S2.** The duration from CR to transplant and maintenance treatement of 6 R/R ALAL-NOS patients.

## Data Availability

Not applicable.
