# Supplementary figures and images for: Efficacy of combination of venetoclax with azacitidine or chemotherapy in refractory/relapse acute leukemias of ambiguous lineage, not otherwise specified
Source: Exp Hematol Oncol. 2021 Sep 16;10:46. doi: 10.1186/s40164-021-00239-w (PMC8447663; doi:10.1186/s40164-021-00239-w)

Table S1


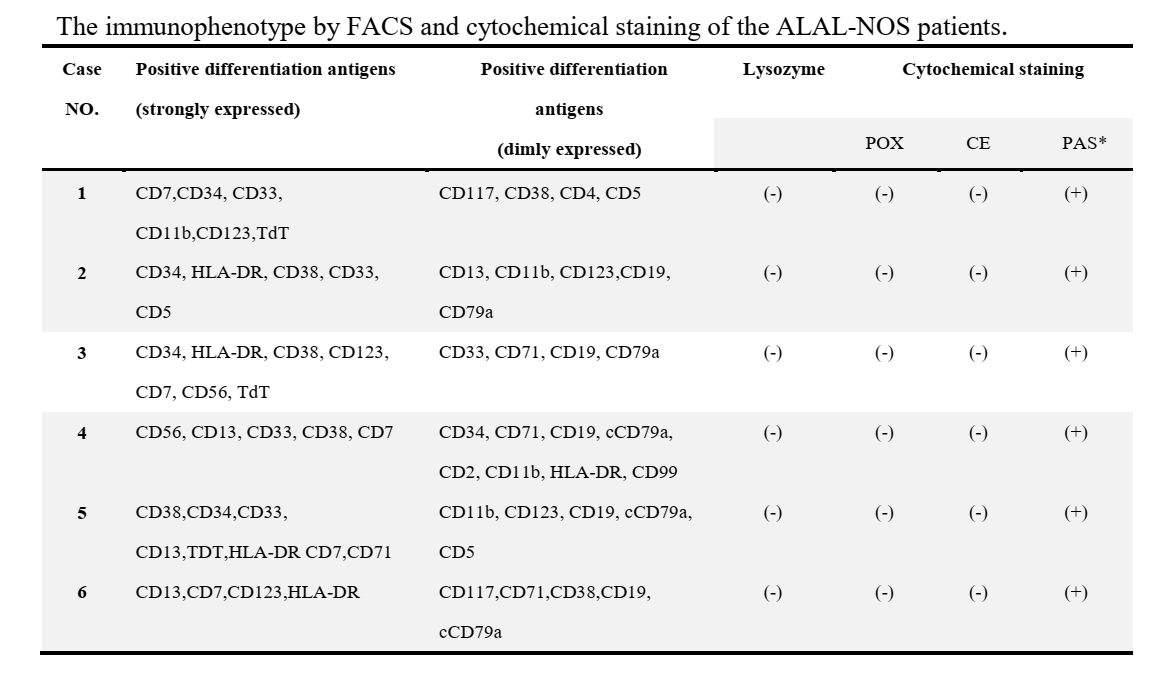


Table S2


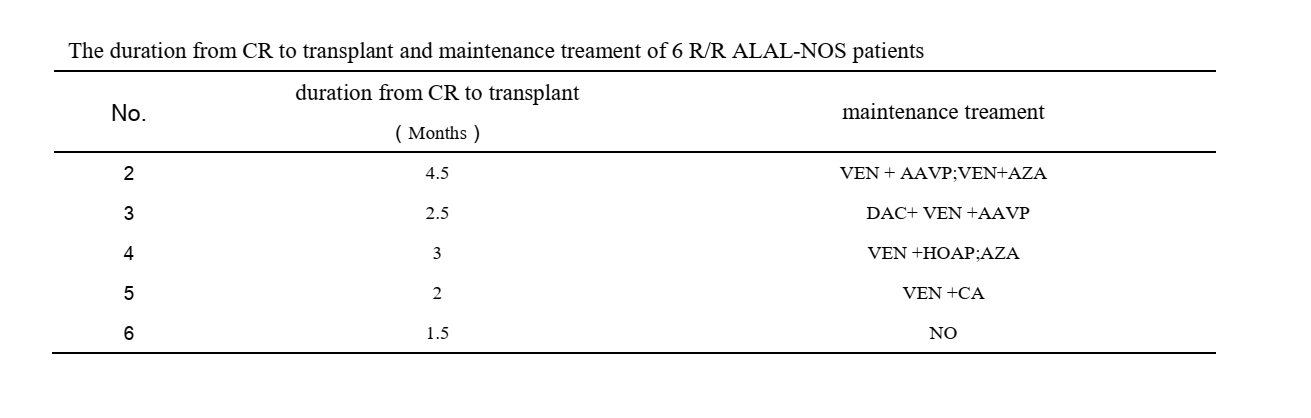

Supplement: Supplementary file 1 — Additional file 1: Table S1. The immunophenotype by FACS and cytochemical staining of the ALAL-NOS patients. Table S2. The duration from CR to transplant and maintenance treatement of 6 R/R ALAL-NOS patients. [file 40164_2021_239_MOESM1_ESM.docx]
